# Mortality associated with HIV-1, HIV-2, and HTLV-I single and dual infections in a middle-aged and older population in Guinea-Bissau

**DOI:** 10.1186/1742-4690-4-85

**Published:** 2007-11-27

**Authors:** Birgitta Holmgren, Zacarias da Silva, Pernille Vastrup, Olav Larsen, Sören Andersson, Henrik Ravn, Peter Aaby

**Affiliations:** 1 Bandim Health Project, Bissau, INDEPTH Network, Guinea-Bissau; 2 Department of Laboratory Medicine, Division of Medical Microbiology/Virology, Lund University, Lund, Sweden; 3 Bandim Health Project, Division of Epidemiology, Statens Serum Institut, Copenhagen, Denmark; 4 Swedish Institute of Infectious Disease Control, Stockholm, Sweden

## Abstract

**Background:**

In Guinea-Bissau HIV-1, HIV-2, and HTLV-I are prevalent in the general population. The natural history of HIV/HTLV-I single and dual infections has not been fully elucidated in this population. Previous studies have shown that combinations of these infections are more common in older women than in men. The present study compares mortality associated with HIV-1, HIV-2, and HTLV-I single and dual infections in individuals over 35 years of age within an urban community-based cohort in Guinea-Bissau.

**Results:**

A total of 2,839 and 1,075 individuals were included in the HIV and HTLV-I mortality analyses respectively. Compared with HIV-negative individuals, adjusted mortality rate ratios (MRRs) were 4.9 (95% confidence interval (CI): 2.3, 10.4) for HIV-1, 1.8 (95%CI: 1.5, 2.3) for HIV-2, and 5.9 (2.4, 14.3) for HIV-1/HIV-2 dual infections. MRR for HTLV-I-positive compared with HTLV-I-negative individuals was 1.7 (1.1, 2.7). Excluding all HIV-positive individuals from the analysis, the HTLV-I MRR was 2.3 (1.3, 3.8). The MRR of HTLV-I/HIV-2 dually infected individuals was 1.7 (0.7, 4.3), compared with HIV/HTLV-I-negative individuals. No statistically significant differences were found in retrovirus-associated mortality between men and women.

**Conclusion:**

HIV-1-associated excess mortality was low compared with community studies from other parts of Africa, presumably because this population was older and the introduction of HIV-1 into the community recent. HIV-2 and HTLV-I-associated mortality was 2-fold higher than the mortality in uninfected individuals. We found no significant differences between the mortality risk for HIV-2 and HTLV-I single infection, respectively, and HIV-2/HTLV-I dual infection. The higher prevalence of retroviral dual infections in older women is not explained by differential retrovirus-associated mortality for men and women.

## Background

Guinea-Bissau constitutes a unique area in which the three major retroviruses HIV-1, HIV-2 and HTLV-I circulate in the general population [1-8]. The natural history of these infections and co-infections is not fully understood in this area. Previously, we have found that various combinations of dual infections of these viruses were more common in women than in men, a trend particularly strong for individuals over 45 years of age[4,5,7,8]. Also in a rural population aged 15 years and over in the same country, we found a strong association between HIV-2 and HTLV-I in women but not in men [7]. Various factors could contribute to these age and gender patterns of retroviral infections. When behavioural factors were included in the analyses our observations were not modified [8]. Differential mortality of retrovirus infections between men and women, compared with retrovirus-negative individuals, could contribute; if retrovirus-infected men have higher mortality than retrovirus-infected women, compared with negative individuals, a higher prevalence of retrovirus infections would be observed among older women. Few studies have investigated mortality associated with HIV-1/HIV-2 dual infection [9,10], and it is unclear whether there are any differences between men and women. Regarding HIV-1 mortality, there is usually no difference between men and women [11-14], although a higher mortality rate for HIV-1-positive men was observed in a professional cohort in Tanzania [15]. HIV-1 mortality studies from community settings in West Africa are scarce [10]. For HIV-2 infections no difference in mortality between men and women was observed either [16-18]. However, none of these studies have examined whether the male-to-female (M/F) mortality ratio differs for HIV-infected and uninfected individuals. In addition, there are few studies addressing HTLV-I-associated mortality in a general population in Africa [4,19].

Hence, we investigated the mortality patterns of HIV-1, HIV-2, and HTLV-I, single and dual infections as well as non-retrovirus infections in the capital Bissau. The objective was also to assess whether the age- and gender-specific patterns of dual retroviral infections could be related to mortality patterns.

## Results

### Participation

A total of 2,839 of the 2,944 individuals were eligible for inclusion in the HIV mortality analyses, and 1,075 of the 1,124 in the HTLV-I analyses. In the HIV analyses, four were not re-identifiable, 15 were excluded due to confusion as to identity or uncertainty about age, and 86 individuals left the study before reaching age 35 (Table 1). The figures for the HTLV-I mortality sample were 1, 11, and 37 individuals (Table 2). A total of 495 (162) individuals moved and 517 (170) died during the follow-up period, numbers in parenthesis indicating individuals in the HTLV-I mortality sample. Tables 1 and 2 display the outcome with regard to HIV/HTLV status and gender. Total follow-up time was 12,283 and 4,022 years for subjects included in the HIV and HTLV-I analyses, with a median follow-up time of 3.8 years (range 0.0–12.4) and 3.0 years (0.0–10.5) respectively. Median age of participants were 53.2 (interquartile range (IQR) = 45.9–61.0), and 49.4 (IQR = 39.2–58.2), respectively.

**Table 1:** Outcome and mortality rates with regard to HIV status, irrespective of HTLV-I status.

	**Negative**		**HIV-1**		**HIV-2**		**HIV-1 + HIV-2 dual**		**Total**
					
	**Men**	**Women**	**Men**	**Women**	**Men**	**Women**	**Men**	**Women**	**All**
Previous HIV result from 1987, 1989, 1990, 1992, 1994 or 1996.	1,250	1,260	10	12	156	243	5	8	2,944
**Total re-identified**	**1,247**	**1,259**	**10**	**12**	**156**	**243**	**5**	**8**	**2,940**
Confusion/age uncertain	1/0	4/0	0/0	0/0	4/1	4/1	0/0	0/0	13/2
Exit study at age <35.	37	43	0	0	2	4	0	0	86
**Total participating in analysis:**	1,209	1,212	10	12	149	234	5	8	**2,839**

Seroconvert during follow-up time	7	9			2	3			
(status after sero-conversion)	(2 HIV-1, 4 HIV-2 1 HIV-1/2)	(HIV-2)			(HIV-1/2)	(HIV-1/2)			

Moved (%*)	198 (16.5)	212 (17.6)	4 (33.3)	1 (8.3)	23 (15.2)	51(21.3)	4 (50)	2 (18.2)	**495 (17.4)**
Died (%*)	229 (19.1)	171 (14.2)	4 (33.3)	3 (25)	42 (27.8)	63 (26.3)	2 (25)	3 (27.3)	**517 (18.2)**
Median follow-up time in years	3.8	3.9	2.5	3.1	3.8	3.7	3.2	2.6	3.8
(range)	(0.0–12.1)	(0.0–12.4)	(0.2–4.3)	(0.4–3.8)	(0.1–11.6)	(0.1–12.0)	(0.3–5.9)	(0.6,4.5)	(0.0–12.4)
Total follow-up time, years	5084	5398	26.9	33.6	648	1039	23.7	29.7	12283
Mortality rate/1000 pyo	45.0	31.7	149	89.3	64.8	60.6	84.3	101	42.1
(95% CI†)	(39.5,51.3)	(27.3, 36.8)	(55.9, 397)	(28.8, 278)	(47.9, 87.7)	(47.3,77.6)	(21.1, 337)	(32.6, 313)	(38.6, 45.9)

Individuals in general urban population cohorts in Guinea-Bissau with an HIV result from any previous survey during 1987–1996 were included in the study. The table shows the outcome of the eligible individuals according to HIV status. Some individuals changed HIV status during follow-up, from HIV-negative to HIV-1 and/or HIV-2-positive, or from HIV-2 to HIV-1/HIV-2 dually positive. This is shown in the middle section of the table.  *% of total number participating according to current HIV status. †CI = confidence interval

**Table 2:** Outcome and mortality rates with regard to HTLV status, irrespective of HIV-status. Individuals from the same cohorts as in table 1 are included, but with an HTLV-1 result from any previous survey

	**Negative**		**HTLV-I**		**Total**
			
	**Men**	**Women**	**Men**	**Women**	**All**
Previous HTLV result from 1990 or 1996.	497	550	20	57	1,124
**Total re-identified with a previous HTLV result**	**496**	**550**	**20**	**57**	**1,123**
Confusion/age uncertain	4/0	5/0	0/0	2/0	11
Exit study at age <35.	21	16	0	0	37
**Total participating in analysis:**	471	529	20	55	**1,075**
					
Moved (%*)	75 (15.9)	80(15.1)	3 (15%)	4 (7.3)	**162 (15.1)**
Died (%*)	79 (16.8)	69 (13.0)	7 (35%)	15 (27.2)	**170 (15.8)**
					
Median follow-up time in years (range)	2.9 (0.0–9.1)	2.9 (0.0–10.5)	3.0 (0.8–7.8)	3.4 (0.8–9.4)	3.0 (0.0–10.5)
Total follow-up time in years	1703	2004	73.4	242	4022
Mortality rate/1000 pyo (95%CI^†^)	46.4 (37.2, 57.8)	34.4 (27.2 43.6)	95.4 (45.5, 200)	61.9 (37.8, 103)	42.3 (36.4, 49.1)

*% of total number participating.

† CI = confidence interval

### General HIV-associated mortality

Sixteen HIV-negative individuals seroconverted during the follow-up period; 13 to HIV-2, 2 to HIV-1, and 1 dual HIV-1/HIV-2 infection. Five HIV-2-positive individuals seroconverted to dual HIV-1/HIV-2 infection. As stated in the statistical methods these seroconversions were accounted for in the analyses.

Among the 517 deaths, 277 occurred among men (MR/1000 pyo = 47.9, 95 percent CI: 42.6, 53.9) and 240 among women (MR = 36.9, CI: 32.5, 41.9). Seven of these deaths occurred among HIV-1-infected (MR = 116, CI: 52.2, 243), 105 deaths among HIV-2-infected (MR = 62.0, CI: 51.4, 75.3) and five among HIV-1 + 2 dually infected (MR = 93.6, CI: 39.0, 225). Overall MRRs, adjusted for current age, current HIV status and sex were 4.9 (2.3, 10.4) for HIV-1, 1.8 (1.5, 2.3) for HIV-2, and 5.9 (2.4, 14.3) for HIV-1/HIV-2 dual infections (Table 3).

**Table 3:** All retrovirus mortality rates and mortality rate ratios

	MR* (95% CI^†^)	**MRR^‡^(95% CI)**
HIV-1	116 (52.2, 243)	4.9^§ ^(2.3, 10.4)
HIV-2	62.0 (51.4, 75.3)	1.8^§ ^(1.5, 2.3)
HIV-1/2 dual	93.6 (39.0, 225)	5.9^§ ^(2.4, 14.3)

HTLV-I all	69.7 (45.9, 106)	1.7^# ^(1.1, 2.7)
HTLV I positive/HIV- negative	77.4 (47.4, 126)	2.3^&^(1.3, 3.8)

* MR = mortality rate per 1000 person years of observation.

† CI = confidence interval.

‡ MRR = Mortality rate ratio.

§Comparing with HIV-negative.

# Comparing with HTLV-I-negative.

& Comparing with HIV and HTLV-I-negative

In a sub-analysis, possible confounding variables were tested such as ethnic group, district, schooling, and working outside home at the time of entry into the study, because this information was not available for all individuals. Only working outside home was independently associated with less risk of dying, the MRR corrected for age and gender being 0.7 (95 percent CI: 0.5, 1.0). The information about work was available for 1707 individuals. Corrected for age, gender, current HIV-status, and work, the HIV-1-associated MRR was 4.1 (95 percent CI: 1.9, 8.7), for HIV-2 the MRR was 1.7 (95 percent CI: 1.3, 2.4), and for dual HIV it was 11.7 (95 percent CI: 4.7, 29.2).

A total of 809 individuals had an HTLV-I status at the time of entry into the study. Adjusting for HTLV-I status did not change the estimates.

### Gender-specific HIV-associated mortality

The mortality rate was higher in HIV-negative men than in women (Table 4), the age-corrected male-to-female (M/F) MRR being 1.4 (95 percent CI: 1.1, 1.7) in HIV-negative individuals.

**Table 4:** Mortality rates (MRs) and mortality rate ratios (MRRs) for HIV, according to age and sex, irrespective of HTLV-I status

	**HIV-negative**	**HIV-1**			**HIV-2**			**HIV-1 + HIV-2**		
				
Age group*	**MR^†^(deaths/pyo)**	**MR^†^(deaths/pyo)**	**MRR^‡^**	**95% CI^§^**	**MR^†^(deaths/pyo)**	**MRR^‡^**	**95%CI^§^**	**MR^†^(deaths/pyo)**	**MRR^‡^**	**95%CI^§^**
**MEN**										
35–44	4.7(4/844)	100(1/10)	21.1	2.4,189	17.9(2/112)	3.8	0.7,20.6	0(0/12.1)		
45–54	20.9(28/1342)	235(2/8.5)	10.3	2.7,47.3	46.9(9/192)	2.2	1.1,4.8	320(2/6.3)	15.3	3.6,64.3
55–64	45.5 (81/1781)	0 (0/4.6)			81.7(18/220)	1.8	1.1,3.0	0 (0/5.3)		
65–74	88.2 (65/737)	268(1/3.7)	3.0	0.4,21.9	124 (13/104)	1.4	0.8,2.6	0 (0/0)		
75+	134 (51/380)	0 (0/0)			0(0/19.9)			0 (0/0)		
**Total:**	**45.0(229/5084)**	**148.8(4/26.9)**	**5.2^#^**	**1.9,14.0**	**64.8(42/648)**	**1.6^#^**	**1.1, 2.2**	**84.3 (2/23.7)**	**4.3^#^**	**1.1,17.3**
**WOMEN**										
35–44	1.9 (2/1040)	0(0/11.3)			33.3(6/180)	17.4	3.5,86.0	97.7(1/10.2)	50.8	4.6,560
45–54	13.3 (18/1346)	227(2/8.8)	17.0	3.9,73.1	19.3(6/311)	1.4	0.6,3.6	57.4(1/17.4)	4.3	0.6,32.1
55–64	31.7 (57/1798)	74.3(1/13.5)	2.3	0.3,16.9	91.8(33/359)	2.9	1.9,4.4	485(1/2.1)	15.3	2.1,110
65–74	63.8 (55/862)	0(0/0)			73.9(11/149)	1.2	0.6,2.2	0 (0/0)		
75+	110 (39/352)	0(0/0)			174(7/40.2)	1.6	0.7,3.5	0 (0/0)		
**Total:**	**31.7(171/5398)**	**89.3 (3/33.6)**	**4.7^#^**	**1.5,14.6**	**60.6(63/1040)**	**2.1^#^**	**1.6,2.8**	**101(3/29.7)**	**7.9^#^**	**2.5,24.9**

*Indicates current age, i.e. individuals can contribute to several age groups.

† MR = mortality rate per 1000 person years of observation.

^‡ ^MRR = Mortality rate ratio, comparing with HIV-negative.

^§^CI = confidence interval.

# Adjusted for age and current HIV status in a Poisson regression, see statistical methods

#### HIV-1 mortality

Comparing HIV-1-positive with HIV-negative individuals, we found the age-corrected MRR to be 5.2 (95 percent CI: 1.9, 14.0) in men and 4.7 (95 percent CI: 1.5, 14.6) in women (test of homogeneity, p = 0.89) (Table 4). When we considered both HIV-1 single infection and dual infections as HIV-1 infection, individuals <45 years of age tended to have stronger excess mortality compared with HIV-negative individuals than individuals > = 45, though the differences were not statistically significant (test of homogeneity, p = 0.41 in men and p = 0.32 in women) (Table 5).

**Table 5:** Mortality rates and mortality rate ratios for HIV, irrespective of HTLV-I status. MRs and MRRs below and above 45 years of age are compared

	HIV-1 and HIV-1 + HIV-2 dually infected*			**HIV-2 single infected^†^**		
		
**Age group**	**Mortality rate/1000 pyo**	**MRR^‡^**	**95% CI^§^**	**Mortality rate/1000 pyo**	**MRR^‡^**	**95% CI^§^**
**MEN**						
<45	45.1(6.4–320)	12.0^#^	1.4,99.7	17.9(4.5,71.6)	4.8^#^	1.0,23.6
> = 45	176(73.2–422)	4.6	1.9,11.1	74.6(54.7,102)	1.5	1.1, 2.2
Test of homogeneity of effect modification of age			*p *= 0.41			*p *= 0.18
**WOMEN**						
<45	46.4(6.5–329)	17.1^#^	2.1,142	33.4(15.0,74.3)	12.3^#^	4.0,38.9
> = 45	120(49.8–288)	5.4	2.2,13.1	66.3(51.1,85.9)	1.9	1.4,2.6
Test of homogeneity of effect modification of age			*p *= 0.32			*p *< 0.005

*Including both HIV-1 single and HIV-1 + HIV-2 dual infections.

^†^Including only HIV-2 single infections.

^‡ ^MRR = Mortality rate ratio, comparing with HIV-negative.

^§ ^CI = confidence interval.

^#^The MRRs are different from those in Table 4 as the HIV-1 single and HIV-1 + HIV-2 dually infected have been grouped together in this Poisson model (see statistical methods)

#### HIV-2 mortality

For HIV-2 the age-adjusted MRR was 1.6 (95 percent CI: 1.1, 2.2) in men and 2.1 (95 percent CI: 1.6, 2.8) in women, test of homogeneity, p = 0.24 (Table 4). Women <45 had higher HIV-2 associated excess mortality than women > = 45 (test of homogeneity, p < 0.005). Though the pattern was the same for men the difference was not statistically significant (test of homogeneity, p = 0.18) (Table 5).

#### HIV-1 + HIV-2 dual mortality

For HIV-1 + 2 dual infection, the age adjusted MRR was 4.3 (95 percent CI: 1.1, 17.3) in men and 7.9 (95 percent CI: 2.5, 24.9) in women, (test of homogeneity, p = 0.51) (Table 4).

### HTLV-I mortality

One individual seroconverted during follow-up. Twenty-two HTLV-I-positive (MR = 69.7, CI: 45.9, 106) and 148 HTLV-I-negative individuals (MR = 39.9, cent CI: 34.0, 46.9) died. Overall age- and sex-adjusted HTLV-I MRR, comparing HTLV-I-positive with HTLV-I-negative individuals, was 1.7 (1.1, 2.7). The age-adjusted MRRs were 1.9 (CI: 0.9, 4.0) and 1.6 (CI: 0.9, 2.8) for men and women respectively (test of homogeneity, p = 0.78) (Table 6). Adjusting for possible confounding variables such as schooling, working outside home, ethnic group, or district did not change the estimates. When all HIV-positive individuals were excluded from the analysis, the MRR for HTLV-I-positive compared with HTLV-I negative individuals was 2.3 (1.3, 3.8) (Table 3).

**Table 6:** Mortality rates (MRs) and mortality rate ratios (MRRs) for HTLV-I according to age and sex, irrespective of HIV status

	**HTLV-I-negative**	**HTLV-I-positive**		
		
Age group*	**MR^†^(deaths/pyo)**	**MR^†^(deaths/pyo)**	**MRR^‡^**	**95%CI^§^**
**MEN**				
35–44	7.0(3/426)	88.2 (1/11.3)	12.5	1.3, 120.3
45–54	29.7(11/371)	0 0/16.0)		
55–64	43.6(20/458)	0 (0/26.5)		
65–74	78.3(25/319)	396(5/12.6)	5.1	1.9,13.2
75+	155(20/129)	144(1/6.9)	0.9	0.1,6.9
**Total:**	**46.4(79/1703)**	**95.4(7/73.4)**	**1.9 ^#^**	**0.9,4.0**
**WOMEN**				
35–44	5.5 (3/545)	99.9 (2/20.0)	18.2	3.0,109
45–54	13.4(5/372)	0 (0/67.0)		
55–64	45.9(28/610)	88.2(9/102)	1.9	0.9,4.1
65–74	64.8(23/355)	54.2(2/36.9)	0.8	0.2,3.6
75+	82.3 (10/122)	121(2/16.5)	1.5	0.3, 6.7
**Total:**	**34.0(69/2004)**	**61.9(15/242)**	**1.6 ^#^**	**0.9,2.8**

*Indicates current age, i.e. individuals can contribute to several age groups.

† MR = mortality rate per 1000 person years of observation.

‡ MRR = Mortality rate ratio, comparing with HTLV-negative.

§CI = confidence interval.

# Adjusted for age in a Poisson regression, see statistical methods

### All retrovirus-associated mortality

We compared mortality for co-infected individuals with mortality for single infected individuals. Among 1,069 individuals with concurrent HIV and HTLV-I results, the MRR for individuals infected only with HIV-1 was 8.9 (95 percent CI: 2.1, 38.6), for HIV-2 single infected it was 2.9 (95 percent CI: 2.0, 4.2), and for HTLV-I single infected 2.3 (95 percent CI: 1.3, 3.8). For HIV-1/HIV-2 dual infections the MRR was 7.0 (95 percent CI: 1.7, 28.7), for HIV-2/HTLV-I dual infections it was 1.7 (95 percent CI: 0.7, 4.3), and for HIV-1/HIV-2/HTLV-I triple infections it was 6.7 (95 percent CI: 0.9, 50.1). All estimates were compared with HIV and HTLV-I-negative individuals. There was no significant difference between MRRs for HIV-2 single infection, HTLV-I single infection and HIV-2/HTLV-1 dual infection.

## Discussion

To our knowledge this is the first study to report comparative mortality rates of HIV-1, HIV-2, and HTLV-I single and dual infections within the same population in a community setting in Africa. The population was antiretroviral treatment naïve at the time of the study; thus these data reflect the natural development of these infections. Generally, the HIV-associated mortality was higher compared with the mortality in non-infected individuals, regardless of the type of HIV infection and of HTLV-I status. The HIV-1-associated mortality was 4 to 5-fold increased compared with the mortality in HIV-negative individuals. This figure is lower than figures reported from community studies in other parts of Africa, where 10–20 times higher risks are frequently found [12,13,20-22], and could be explained by the differences in the age distribution between our sample and studies from other areas including mainly younger adults [12,13,20,21]. Reports on HIV-1-associated mortality from general populations in West Africa are scarce [10]. The fact that HIV-1 was introduced recently into Guinea-Bissau and that the HIV-1 epidemic presumably is still in its early phase could also contribute to the lower relative mortality compared with the more high prevalent areas. Another possible explanation could be variations in disease progression between HIV-1 subtypes as reported from Senegal [23]. In West Africa, subtype A seems to be most prevalent [24-26], and studies have indicated that an A/G recombinant form is the most common in West Africa [26-28], in particular in Guinea-Bissau [28].

For HIV-2 we found 1.5 to 2-fold increased mortality rates. This is comparable with previous observations from the same urban [17] and rural general populations [16]. The MRR for dual HIV-1/HIV-2 infection in our study was more equivalent to the risk we found for HIV-1 and could reflect that the clinical outcome of HIV-1 and HIV-2 dual infection may resemble HIV-1 more than HIV-2 [29-32]. However, there were few dually infected in the study, and larger studies are needed to determine whether these trends are reproducible. Few other community studies have addressed mortality related to HIV-1 and HIV-2 dual infection [10].

HTLV-I-associated mortality was about 2-fold increased compared with HTLV-I-negative mortality. The effect was particularly strong when excluding the HIV-positives from the analysis. A previous follow-up of individuals aged ≥ 50 years in this population did not identify any significant effect of HTLV-I on survival [4]. A larger number of participants and a lower mean age in the present study may explain this difference. In the rural area of Guinea-Bissau, a 7-year follow-up of 285 HIV-2-positive and HIV-2-negative individuals found no increased mortality for HTLV-I. However, mortality was associated with increased HTLV-I proviral load [19]. Theoretically contracting HIV during follow-up could contribute to higher mortality if the course of a newly acquired HIV infection were rapid. The few HIV seroconversions during follow-up that we know of were adjusted for in the analyses. The mortality risk in the present study is slightly higher than the 1.5–2 fold increased HTLV-I mortality found in high prevalent areas such as southern Japan [33-35]. Diseases usually associated with HTLV-I infection are Tropical spastic paraparesis/HTLV-I-associated myelopathy (TSP/HAM) [36,37] and adult T-cell leukemia (ATL) [38,39] and occur in 3–4 percent of HTLV-I-infected individuals [40-42]. There are no data regarding the prevalence and incidence of these diseases from the area but it seems unlikely that these would explain all the excess mortality. The high incidence of Tuberculosis (Tb) in this community [43] might contribute to this increased HTLV-I-associated mortality. Increased prevalence of HTLV-I in Tb patients compared with healthy individuals has been reported from Brazil [44]. It could also reflect other causes of disease and death that have so far not been linked to HTLV-I. In the subset of data where concurrent HIV and HTLV-I result was available, we found no significant difference in the mortality risk for HIV-2/HTLV-I dual infection and the mortality risk for HIV-2 and HTLV-I single infection respectively. Further follow-up studies of HTLV-I single and HTLV-I/HIV dually infected individuals are needed, as are studies identifying diseases associated with HTLV-I infection in this area.

We have previously found that dual retroviral infections are more common in women than in men, and that the prevalence increases with age in women, while it decreases with age in men [8]. In the present study we could not identify any gender differences in HIV and HTLV-I-associated mortality explaining our previous observation. However, according to Table 1 more HIV-1 and dual HIV-1/HIV-2 positive-men than women moved during follow-up compared with HIV-negative individuals. This was also seen for HTLV-I-positive men. The individuals who moved were censored on the date of migration. Thus informative censoring could occur in our analyses if the reason for having migrated was associated with a higher or lower risk of dying. The HIV-1-positive men who moved were younger than those who did not move (data not shown) so this could potentially bias our results. However, if higher HIV-associated mortality in men would explain the higher prevalence of dual infections among women, this may rather be due to HIV-2 and dual infection-related mortality, viewing the epidemiological features of the viruses in this population. HIV-2 has been endemic in the country for decades, while HIV-1 was introduced more recently, as illustrated by incidence data which did not show any sero-conversion to HIV-2 in individuals already infected with HIV-1 or HTLV-I [8]. For HIV-2 there was no difference in loss to follow-up between men and women that could bias the results with regard to HIV-2-associated mortality. The additional risk of dying associated with HIV was higher in younger individuals than in older. Though only statistically significant for HIV-2 in women, the trend was seen for both men and women regarding both HIV-1 and HIV-2, probably reflecting higher background mortality in older individuals. A comparison between age groups is, however, difficult to interpret as we have no information on when the individuals contracted their retrovirus infections. But also in previous follow-up studies of HIV-2 [16,17] HIV-2-associated mortality was found to be higher among younger individuals.

The present data indicate that there is no differential mortality between men and women explaining the higher prevalence of dual retroviral infections in older women, nor could it be explained by behavioural factors [7,8]. This might provide further indirect support for the hypothesis of an enhanced biological susceptibility to retroviral infections of older women compared with men [45-47]. The major change around 45 years of age corresponds to the pre-menopausal and menopausal age. Alterations in female hormones and the associated histological changes could induce changes in the immunological defence of the genital tract (reviewed in [48]). In animal models of HIV infection it has been demonstrated that susceptibility to SIV is related to hormonal status [49-51]. Further epidemiological studies on incidence patterns as well as biological studies are warranted to determine the generality and mechanisms of this trend towards increased HIV prevalence among older women. This trend could have major public health implications with the longer survival of HIV-infected individuals, and older women might constitute a vulnerable group to be emphasized in future HIV/AIDS prevention control programmes.

## Conclusion

HIV-1-associated mortality was 4–5-fold, and HIV-2-associated mortality was 2-fold higher than HIV-negative mortality. The mortality risk for HIV-1/HIV-2 dual infection was equivalent to the risk for HIV-1. HTLV-I MRR was 2.3(1.3–3.8) when HIV-positive individuals were excluded from the analysis. There was no difference between men and women explaining the previous finding of higher prevalence of dual infections in older women.

## Methods

### Study area and population

The study area is divided into three adjacent districts, Bandim 1, Bandim 2, and Belem. A community-based cohort, established in 1987 [2,17,52] and including individuals >15 years of age from 100 randomly selected houses, was extended in 1995 to encompass an additional 212 randomly selected houses [3]. Surveys in this cohort were performed in 1987, 1989, 1992, and 1996 [2,3,17,52]. A second cohort, established in 1990, included all individuals aged 50 years and over resident in Bandim 1 and 2 based on a general population census in 1986–1987 and was extended in 1994 to encompass all individuals aged ≥ 50 years from the entire study area [4,53]. Surveys in this cohort were performed in 1990 and 1994. Laboratory methods have been described elsewhere [2-4,17,52,53]. Subjects from both cohorts aged 35 years and over at the beginning of 1998 were included in a survey of HIV and HTLV infections in 1998–2000. The reason for this was to assess the dynamics of retroviruses in older age groups, as previous studies from the area had suggested a higher susceptibility to HIV-2 in women older than 45 years of age [17,54]. The study included 3,560 subjects [8]. All individuals who had an HIV or HTLV status from any of the previous surveys during 1987–1996 (N = 2,944 and N = 1,124 for HIV and HTLV respectively) were enrolled in the present mortality analyses (Tables 1 &2).

Date of entry into the present study was the date corresponding to the date of first participation ever in a screening. Information on vital status was obtained during the study performed in 1998–2000. If a participant had died or moved, a family member or a neighbour provided this information. Date of exit from the study ranged from 1987 to 2000.

### Statistical methods

Mortality rates (MRs) were defined as the number of deaths per 1000 person years of observation (pyo). Date of exit for survivors was defined as the date during the follow-up study in 1998–2000 at which the subject was encountered alive. For those who had moved or died, follow-up time was censored at the midpoint of the month when only the month for migration/death was given, and at the midpoint of the year when only the year was given. If the date of migration or death was missing (N = 156), follow-up time was censored at the midpoint between date of entry into the study and the date on which the information about migration or death was obtained, because more specific information was not available. Poisson regression was used in the mortality analyses giving mortality rate ratios (MRRs) and their 95 percent CIs, controlling for current age during follow-up (35–44, 45–54, 55–64, 65–74, 75+ years) and current HIV status [55]. This was done for HIV-1, HIV-2, and HIV-1 + HIV-2 dual infections in the same model. Date of first participation in any screening or date of turning 35 years old, was used as date of entry in analyses, which ever came last. To account for possible seroconversion during the follow-up period, the dataset records for sero-converters were split into episodes before and after seroconversion, i.e. at date of first positive sample. To test if men and women had equal mortality risk associated with retrovirus infections, we tested the interaction between gender and infection in the Poisson regression, i.e a homogeneity test. To assess whether age had an effect on the HIV-related excess mortality, we performed a test of homogeneity with age divided into below and over 45 years of age. In this analysis the HIV-1 infection group includes both HIV-1 and HIV-1 + HIV-2 dual infections since there were too few HIV-1 cases to permit further division into age groups. Analyses were performed in Stata version 8.

### Ethical considerations

A protocol of the study was approved by The Central Scientific-Ethical Committee of Denmark and The Ministry of Public Health in Guinea-Bissau. Informed verbal consent was obtained from every subject prior to interview and blood sample. Antiretroviral treatment was not available in the country at the time of the study. Participants had access to free medical consultation and basic treatments, HIV counselling and information and free condoms.

## Competing interests

The author(s) declare that they have no competing interests.

## Authors' contributions

The study was planned by BH and PA and executed by BH and ZS. OL and PA carried out the previous cohort studies, and SA was responsible for the laboratory test strategies. PV and HR were responsible for the statistical analyses. BH carried out the data management and data analyses and wrote the first draft. All authors contributed with interpretation of the data and to the final version of the paper.
